# Populations and Dynamics of Guanine Radicals in DNA strands—Direct versus Indirect Generation

**DOI:** 10.3390/molecules24132347

**Published:** 2019-06-26

**Authors:** Evangelos Balanikas, Akos Banyasz, Gérard Baldacchino, Dimitra Markovitsi

**Affiliations:** 1 LIDYL, CEA, CNRS, Université Paris-Saclay, F-91191 Gif-sur-Yvette, France; vangelis.balanikas@cea.fr (E.B.); akos.banyasz@ens-lyon.fr (A.B.); gerard.baldacchino@cea.fr (G.B.); 2 Univ Lyon, ENS de Lyon, CNRS UMR 5182, Université Claude Bernard Lyon 1, Laboratoire de Chimie, F-69342 Lyon, France

**Keywords:** DNA, guanine quadruplexes, radicals, electron holes, oxidative damage, photo-ionization, time-resolved spectroscopy, inhomogeneous reactions

## Abstract

Guanine radicals, known to be involved in the damage of the genetic code and aging, are studied by nanosecond transient absorption spectroscopy. They are generated in single, double and four-stranded structures (**G**-quadruplexes) by one and two-photon ionization at 266 nm, corresponding to a photon energy lower than the ionization potential of nucleobases. The quantum yield of the one-photon process determined for telomeric G-quadruplexes (**TEL25/Na^+^**) is (5.2 ± 0.3) × 10^−3^, significantly higher than that found for duplexes containing in their structure GGG and GG sequences, (2.1 ± 0.4) × 10^−3^. The radical population is quantified in respect of the ejected electrons. Deprotonation of radical cations gives rise to (**G-**H1)^•^ and (**G**-H2)^•^ radicals for duplexes and **G**-quadruplexes, respectively. The lifetimes of deprotonated radicals determined for a given secondary structure strongly depend on the base sequence. The multiscale non-exponential dynamics of these radicals are discussed in terms of inhomogeneity of the reaction space and continuous conformational motions. The deviation from classical kinetic models developed for homogeneous reaction conditions could also be one reason for discrepancies between the results obtained by photoionization and indirect oxidation, involving a bi-molecular reaction between an oxidant and the nucleic acid.

## 1. Introduction

**Figure 1:**
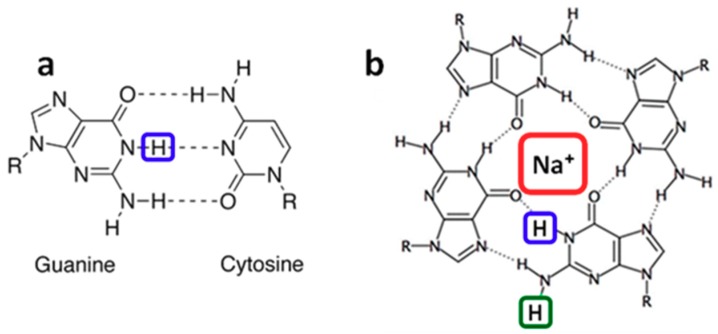
In double helices guanine is paired to cytosine (**a**). Guanines may also self-associate forming a tetrad (**b**) which is the building block of **G**-quadruplexes. (**G**-H1)^•^ and (**G**-H2)^•^ radicals correspond to the transfer of the protons encased in blue and green, respectively, toward the aqueous solvent. Na^+^ encased in red represents a sodium ion located in the central cavity of the G-quadruplex.

Guanine (**G**) radicals are major actors in the oxidatively generated damage to the genetic code [1]. The reason is that **G** is the nucleobase with the lowest oxidation potential [2]. Therefore, electron holes (radical cations) created on other nucleobases of a DNA helix, may reach **G** sites following a charge transfer process and, subsequently, undergo irreversible chemical reactions [3,4,5,6,7]. Various reaction mechanisms have been determined [8,9]. Some of them, such as formation of the well-known oxidation marker 8-oxo-7,8-dihydro-2′-deoxyguanosine (8-oxodGuo), involve directly the radical cation (**G**)^•+^. However, this charged species is prone to loss of a proton, giving rise to deprotonated radicals, labeled (**G**-H1)^•^ [10,11,12,13,14] and (**G**-H2)^•^ [15,16,17,18,19] (Figure 1), depending on the position from which the proton is lost (Figure 1). Further reactions implicate deprotonated radicals [8]. Accordingly, the fraction of (**G**)^•+^ that undergoes deprotonation, as well as the lifetime of the various radicals are expected to play a pivotal role in the relative yields of the final reaction products.

Two different approaches, based on time-resolved techniques, have been used in order to characterize the dynamics of **G** radicals. On the one hand, (**G**)^•+^ are formed directly by photoionization [10,18,19,20,21,22,23,24,25,26]. On the other, they are created in an indirect way via a charge transfer reaction requiring mediation of an external oxidant. In turn, the latter may be generated either by laser [17,27,28,29] or electron pulses [11,13,15,16,30]. During the past few years, important discrepancies started to appear in the reported lifetimes of the **G** radicals. For example, indirect oxidation using sulfate ions (SO_4_^•−^) reported that base-pairing induces a faster decay of (**G**-H1)^•^ on the ms time-scale [28]. In contrast, the lifetimes found for (**G**-H1)^•^ by direct photoionization increase in the following order: single strand, double strand, four-stranded structure (**G**-quadruplex) [18,24,26]. More surprisingly, while one indirect study of **G**-quadruplexes reported that radical cations decay with a lifetime of 0.1 ms giving rise to (**G**-H1)^•^ radicals [29], another study, using exactly the same oxidant, showed that (**G**)^•+^ deprotonation in **G**-quadruplexes, occurring on the µs time-scale, gives rise to (**G**-H2)^•^ [17]. This was explained by the participation of the hydrogen in position 1 to a hydrogen bond (Figure 1) [17]. The latter conclusion was supported by our direct photoionization studies, which, in addition, found that (**G**-H2)^•^ → (**G**-H1)^•^ tautomerisation takes place on the ms time-scale [18,19].

The above mentioned discrepancies appear by comparing results obtained for the same secondary structure but different base sequences. However, it is also reported that the base sequence may affect radical dynamics. This is the case of (**G**)^•+^ in four-stranded structures [17,18,19] and of deprotonated adenine radicals in duplexes [24]. Therefore, it is important to explore if the two approaches used for the study of radical dynamics agree when experiments are performed for exactly the same system. This is one objective of the present work.

Our study was performed by nanosecond laser photolysis and used the direct photoionization approach with excitation at 266 nm. We focused on three different types of DNA structures whose study by the indirect approach is well described [28,29]:

➢ two single strands composed of 30 bases
  **S1:** 5′-CGTACTCTTTGGTGGGTCGGTTCTTTCTAT-3′, and
  **S2:** 3′-GCATGAGAAACCACCCAGCCAAGAAAGATA-5′,
➢ the duplex **D** formed by hybridization of **S1** with its complementary strand **S2**, and
➢ the monomolecular **G**-quadruplex formed by folding of the human telomeric sequence 5′-TAGGG(TTAGGG)_3_TT-3′ in the presence of Na^+^ ions, abbreviated as **TEL25/Na^+^**.

The second objective of our study is to examine the extent to which the dynamics of **G** radicals are affected by the base sequence within a given secondary structure (single-, double- or four-stranded). To this end, the present results were compared with those obtained by us previously following the same methodology for a single strand corresponding to the human telomer repeat 5′-TTAGGG-3′ [18], a duplex composed of the guanine-cytosine pairs in alternating sequence **GC_5_** [26], another human telomer **G-**quadruplex formed by a somewhat shorter sequence, 5′-GGG(TTAGGG)_3_-3′ in the presence of Na^+^ ions (**TEL21/Na^+^**) [18] and a tetramolecular G-quadruplex formed by association of four TGGGGT strands (**TG4T)_4_/Na^+ ^ [19]**.

For **D** and **TEL25/Na^+^**, the probability that **G** radicals generated upon direct absorption of single photons with energy lower than the **G** ionization potential was also examined. This unexpected mono-photonic ionization at long wavelengths, suggested by a few authors [31,32,33], has been evidenced recently by concomitant quantification of ejected electrons and generated radicals [18,19,24,26]. It was further supported by the detection of the well-known oxidation marker 8-oxo-7,8-dihydro-2′-deoxyguanosine (8-oxodGuo) in solutions of purified genomic DNA [34] and telomeric **G**-quadruplexes [18] irradiated by continuous light sources at wavelengths ranging from 254 to 295 nm.

## 2. Results and Discussion

### 2.1. Methodology: Advantages and Limitations

**Figure 2:**
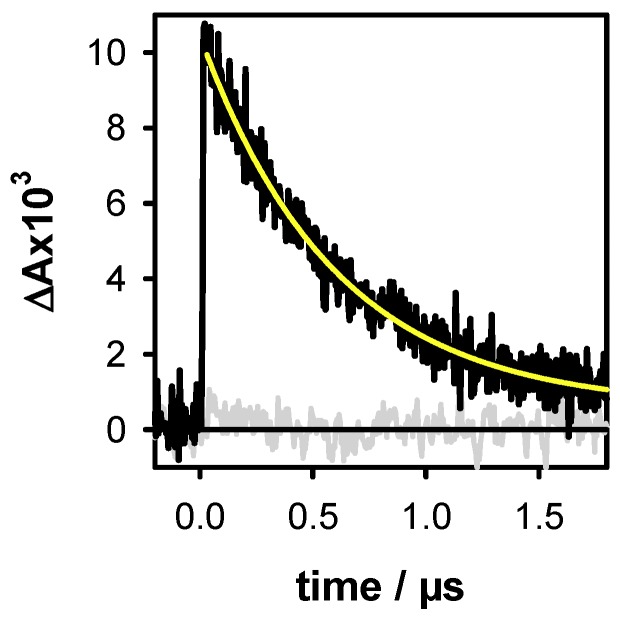
The transient absorption signals recorded at 700 nm for the duplex **D** (black) and the buffer alone (grey) with an excitation intensity of 2 × 10^6^ W cm^−2^. The yellow line represents the fit with a mono-exponential function A_0_ + A_1_exp(−t/τ_1_). Within the precision of our measurements, the intensity of the signals at 720 nm and 700 nm are same. As the latter are less noisy, the electron concentration was systematically determined at this wavelength.

A key point in our methodology is that the DNA solution does not contain any additive besides the phosphate buffer. In addition, electrons are ejected at zero-time in respect of the time resolution of the setup which is ~30 ns. At this time, the ejected electrons have been already hydrated [35]. Under the latter configuration, they exhibit a broad absorption band peaking at 720 nm with a molar absorption coefficient ε of 19,700 mol^−1^ L cm^−1^ [36]. With respect to this property, they can be quantified. For better precision, their decay was fitted with a mono-exponential function A_0_ + A_1_exp(−t/τ_1_) (Figure 2). Subsequently, the A_1_ value, associated to ε, provides the initial concentration of the hydrated ejected electrons [e_hyd_^−^]_0_. In such an experiment, electrons may originate not only from DNA photoionization, but also from two-photon ionization of water. In order to avoid the latter process, which precludes quantitative correlation between ejected electrons and generated radicals, weak excitation intensities (≤2 × 10^6^ W cm^−2^) were used. Under these conditions, no hydrated electrons were detected for the aqueous solvent alone (Figure 2). Moreover, electrons may react with nucleic acids [37]. However, this unwanted effect is prevented because the hydrated electrons are scavenged by the phosphate groups of the buffer [38], which are present in much higher concentrations than the DNA multimers.

An important drawback of radical generation by direct photoionization is that, in the same time, a series of photoproducts, possibly involving reaction intermediates, are formed [39]. The spectra of such species may overlap with those of radicals, determined after 2 µs, when the hydrated electrons have disappeared. This is, in particular, the case of pyrimidine (6-4) pyrimidone photoproducts (64PPs) formed following reactions between two pyrimidines [40,41], as well as adenine-adenine [42] and adenine-thymine dimers [43,44,45,46,47] and their reaction intermediates [25,48]. All these compounds absorb in the 300 to 400 nm range, exactly where the absorption of **G** radicals is particularly intense. Fortunately, **G** radicals exhibit additional characteristic peaks in the visible spectral domain, thus allowing their identification and quantification.

Finally, a key condition in our methodology is to avoid exciting DNA multimers that have been altered as a result of either photoionization or other photochemical reactions. This is achieved by using a large quantity of solution, which makes such experiments both slow and expensive. Typically, 40 mL of **D** or **TEL25/Na**^+^ solutions are needed for recording a transient absorption spectrum over a single time scale. Considerably larger quantities are required in the case of **S1** and **S2** because the yield of dimeric photoproducts is much higher in single strands [49,50,51]. Therefore, the study of single strands was limited to radical dynamics.

More details on the experimental protocols are given in the Materials and Methods Section.

### 2.2. One- and Two-Photon Ionization

Electron ejection upon 266 nm laser excitation of nucleic acids, provoked by two-photon ionization has been exploited to study oxidative damage to DNA [52,53]. In this study, we tried to keep as low as possible the contribution of the two-photon process but without completely eliminating it, otherwise the transient absorption signals stemming from both the hydrated electrons and the radicals become too weak to be observed. In order to disentangle between one and two-photon effects, the laser intensity was varied and at each step, we determined [e_hyd_^−^]_0_. Subsequently, the ionization curve was obtained by plotting [e_hyd_^−^]_0_/[hν] as a function [hν]. The latter quantity represents the concentration of absorbed photons per pulse in the probed volume of the studied solution. The experimental points are fitted with the linear model function [e_hyd_^−^]_0_/[hν] = φ_1_ + α[hν]. The intercept on the ordinate provides the one-photon ionization quantum yield φ_1_, while the slope is proportional to the two-photon ionization yield φ_2_, which depends on the laser intensity, φ_2_ ∝ α[hν].

**Figure 3:**
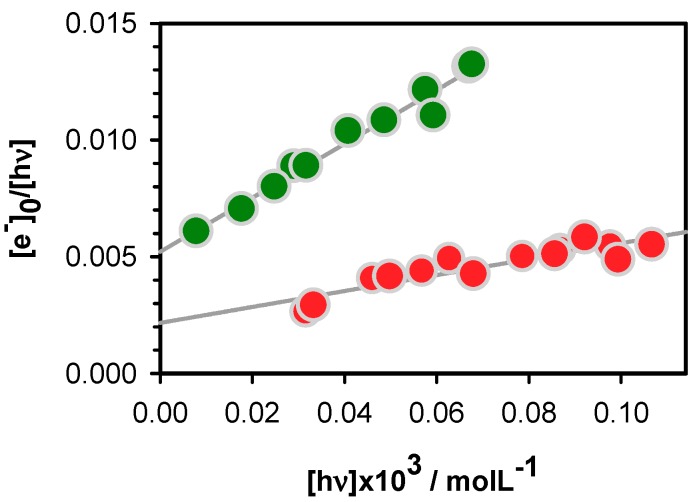
The ionization curves obtained for the duplex **D** (red) and the G-quadruplex **TEL25/Na^+^** (green); [e_hyd_^−^]_0_ and [hν] denote, respectively, the zero-time concentration of hydrated ejected electrons and the concentration of absorbed photons per laser pulse. Experimental points (circles) are fitted with the linear model function [e_hyd_^−^]_0_/[hν] = φ_1_ + α[hν] (grey).

The ionization curves obtained for **D** and **TEL25/Na^+^** are shown in Figure 3. The φ_1_ determined for the duplex is (2.1 ± 0.4) × 10^−3^, while a much higher value, (5.2 ± 0.3) × 10^−3^, is found for the **G**-quadruplex. The higher propensity of **G**-quadruplexes to undergo electron detachment upon absorption of single photons at 266 nm is in line with previously reported results [18,19,24,25,26]. However, in addition, the present work brings to light some subtle differences.

In the case of duplexes, electron detachment is facilitated by the occurrence of one GG and one GGG sequences, for a total of thirty base pairs, composing **D**. As a matter of fact, a φ_1_ value of (1.2 ± 0.2) × 10^−3^, was found for the duplex **GC_5_** [26] while those determined for alternating and homopolymeric AT duplexes amount to (1.3 ± 0.2) × 10^−3^ and (1.5 ± 0.3) × 10^−3^, respectively [24,25]. This is in line with previous findings that the oxidation potential of G is decreased upon stacking, rendering GG and GGG triplets traps [54,55] for hole transfer [7,56,57,58] and preferential sites for redox reactions [59].

Considering the above base sequence effect found for duplexes, it is understandable that telomeric G-quadruplexes, composed of four interconnected GGG stacks, exhibit more efficient one-photon ionization. However, our results show that not only GGG stacks play a role in this process. The φ_1_ value determined for **TEL25/Na^+^** is slightly higher compared to that of **TEL21/Na^+^** (4.5 ± 0.6) × 10^−3^. The difference in the base sequence of these systems is the presence of two flanking groups TT and TA in **TEL25/Na^+^**. These flanking groups do not participate neither to tetrad nor to loop formation.

### 2.3. Radicals in Single and Double Strands

**Figure 4:**
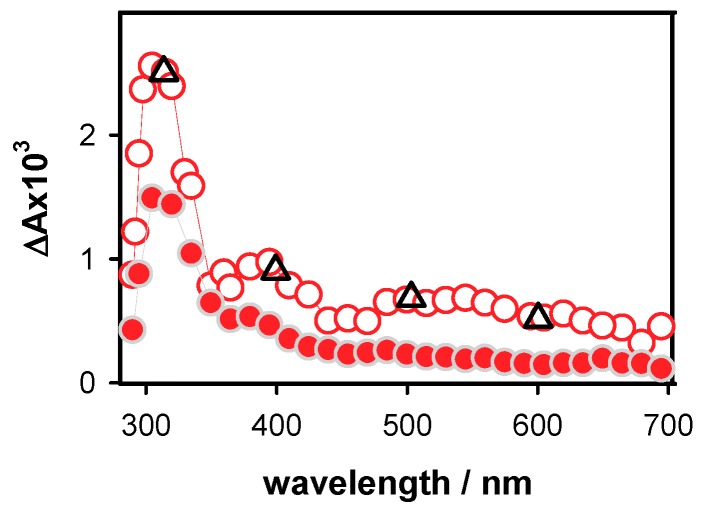
The differential absorption spectra determined for the duplex **D** at 5 µs (empty circles; average ΔA from 3 to 7 µs) and 10 ms (full circles; average ΔA from 8 to 12 ms). The triangles denote relative intensities of the 5 µs spectrum obtained using oxidation by SO_4_^•−^ [28].

The transient absorption spectrum obtained for **D** at 5 µs (Figure 4) resembles closely that of the deprotonated (**G**-H1)^•^ radicals [21]. As discussed in the literature [28,60], deprotonation of guanine radical cations in duplexes may proceed by the transfer of a hydrogen atom to either the cytosine or the aqueous solvent. The spectra of these two deprotonated guanine radicals were computed by quantum chemistry methods for a short duplex composed of two guanine-cytosine pairs in alternating sequences (Figure 6b in reference [26]). It appeared that only the transfer of the proton to the aqueous solvent induces a long red tail in the radical absorption spectrum. Quite recent calculations performed for a guanine-cytosine pair using a larger basis set [61] showed the existence of a weak intensity band between 600 and 650 nm for (**G**-H1)^•^ radical. This feature can be distinguished in the **D** spectrum at 5 µs.

The radical concentration at 5 µs, determined from the differential absorption at 500 nm and the molar absorption coefficient reported for the corresponding monomeric radical (1500 mol^−1^ L cm^−1^) [10], is 4.8 × 10^−7^ mol L^−1^. This value is quite close to the initial electron concentration [e_hyd_^−^]_0_, determined for the same excitation energy (5.1 × 10^−7^ mol L^−1^). The 12% difference falls in the experimental error bar, so that it cannot be excluded that the somewhat lower concentration of radical is due to a reaction taking place at shorter times.

**Figure 5:**
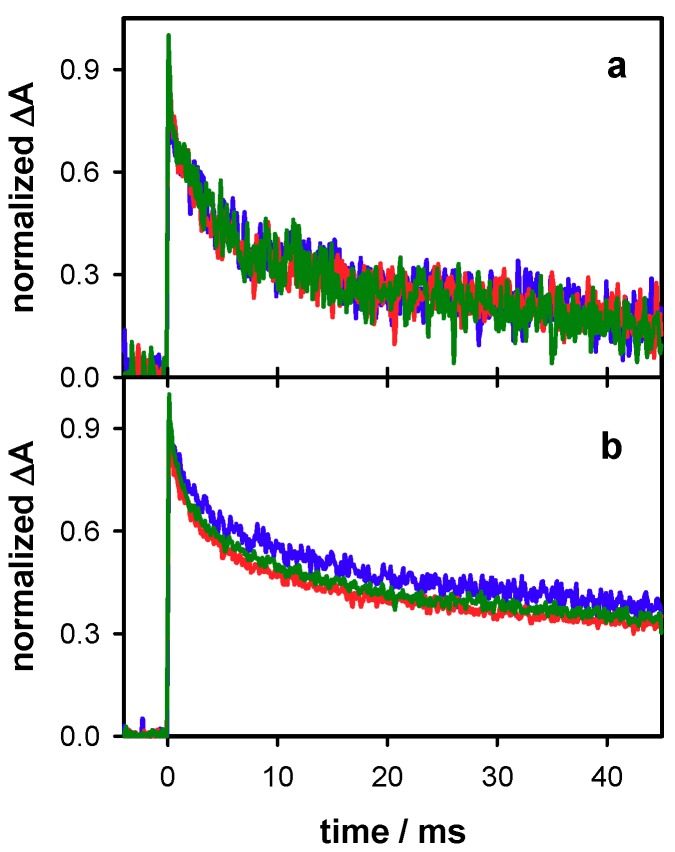
The normalized transient absorption signals recorded for the duplex **D** at 500 (**a**) and 305 nm (**b**) for excitation energies of 4 mJ (blue), 6 mJ (green) and 7 mJ (red), corresponding to decreasing φ_1_/φ_2_ ratios.

At longer times, the relative intensity of the UV band, in respect to the absorption in the visible spectral domain increases, suggesting contribution of photoproducts appearing on the ms time scale. For example, thymine 64PPs are formed within 4 ms [41]. The coexistence of radicals and photoproducts is also reflected in the dependence of the decays recorded on the ms time scale as a function of the laser intensity (Figure 5). Those at 500 nm remain unchanged (Figure 5a), showing that the dynamics of radicals formed by one- or two-photon ionization is the same. However, dimers are generated by one-photon processes, thus their relative concentration is higher at low excitation intensities. **S1** and **S2** exhibit similar behavior in this respect but, as single strands are more prone to dimerization reactions compared to duplexes [49,50,51], the effect on the 305 nm decay is much stronger. An example is given in Figure S1.

**Figure 6:**
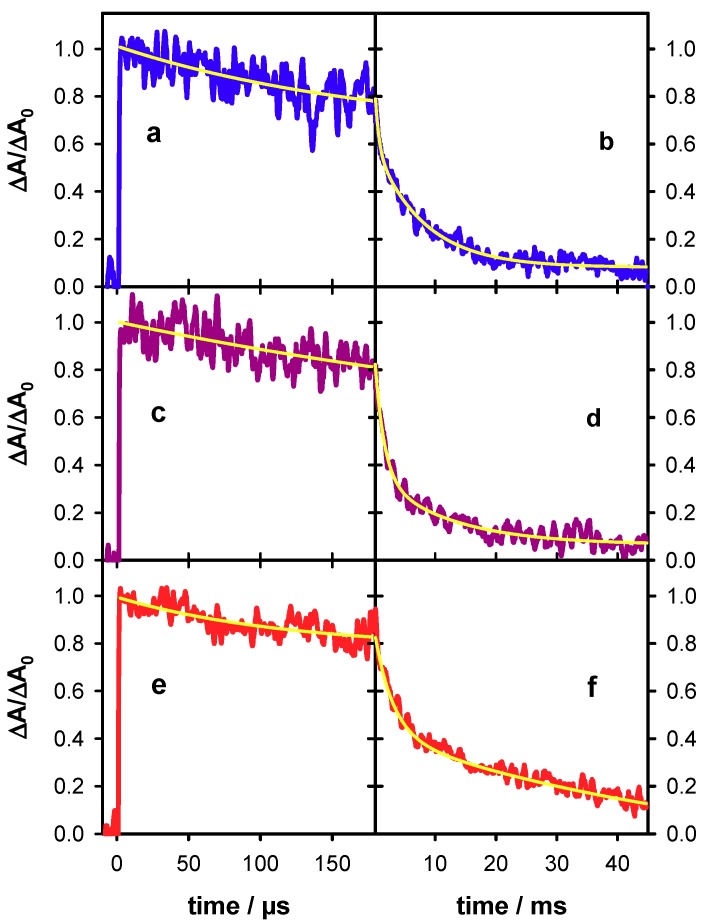
Transient absorption traces recorded for the single strand **S1** (blue; (**a**,**b**)), the single strand **S2** (violet; (**c**,**d**)) and the duplex **D** (red; (**e**,**f**)) at 500 nm. Yellow lines correspond to fits with mono-exponential (**a**,**c**,**e**) and bi-exponential (**b**,**d**,**f**) functions. For all signals, the absorbance at 2 µs (ΔA_0_) was normalized to 1.

The decays recorded at 500 nm over two time-scales for **S1**, **S2** and **D** are shown in Figure 6. They have been fitted with exponential functions and the absorbance at 2 µs has been normalized to 1. For all three systems, an absorbance loss of about 20% was observed within the first 150 µs while at 45 ms only 8% of the initial absorbance persists for **S1** and **S2** and 12% for **D**. The time needed for the signal to decrease by a factor of 2 (t_1/2_) is 1.8 ms and 2.2 ms, respectively, for **S1** and **S2** and significantly longer (4 ms) for **D**. A lengthening of t_1/2_ from 1 to 4 ms was also found upon base-pairing of adenine tracts [24].

**Figure 7:**
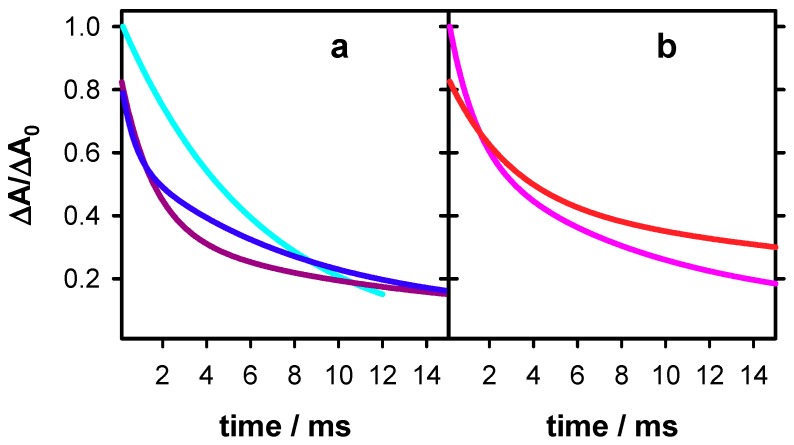
Dynamics of deprotonated guanine radicals observed at 500 nm in single (**a**) and double (**b**) strands. **S1** (blue), **S2** (violet), TTAGGG (cyan; data from reference [18]), **D** (red) and GC_5_ (pink; data from reference [26]). For clarity, only the fitted functions of the transient absorption signals are shown. For all signals, the absorbance at 2 µs (ΔA_0_) was set equal to 1.

It is interesting to compare the dynamics of guanine radicals determined in the present work with those of two other systems studied previously by the same methodology: TTAGGG [18] and **GC_5_** [26]. This is illustrated in Figure 7, where the dynamics at 500 nm between 0.15 and 15 ms are shown. For clarity, only the fitted functions are presented. It is noted that those of TTAGGG and **GC_5_** remain constant between 2 µs and 0.15 ms. It appears that, although for all systems the most important part of the absorbance decays within this time-scale, the decay patterns are specific to each system.

### 2.4. Radicals in G-Quadruplexes

**Figure 8:**
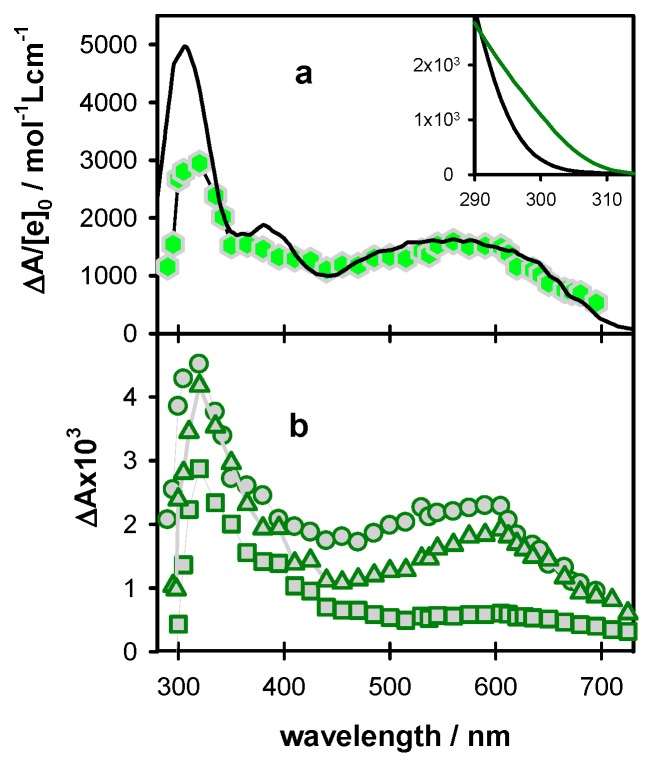
Differential absorption spectra determined for **TEL25/Na^+^** at 3 µs ((**a**); hexagons; average ΔA from 2 to 4 µs), 5 µs ((**b**); circles; average ΔA from 3 to 7 µs), 0.5 ms ((**b**); triangles; average ΔA from 0.3 to 0.7 ms) and 10 ms ((**b**); squares; average ΔA from 8 to 12 ms). The black line in (**a**) is a linear combination of the spectra corresponding to the radical cation (45%) [10] and the (**G**-H2)^•^ radical (55%) [16] of monomeric guanosine, considered with their ε values. In the inset, the steady-state absorption spectra of dGMP (black) [62] and **TEL25/Na^+^** (green; see also Figure S5) are shown. The ε is given per base.

The differential absorption spectra determined for **TEL25/Na^+^** exhibit important variations as a function of time on the visible spectral domain, where **G** radicals are expected to absorb (Figure 8). The spectrum at 3 µs it is characterized by a very broad absorption band, indicating the presence of at least two species. At 0.5 ms, the differential absorbance has decreased between 400 and 600 nm while it has been hardly altered at longer wavelengths. A rather symmetrical band peaking at 600 nm was observed. The latter resembles that of monomeric (**G**-H2)^•^ radicals [10,16]. As found by Su and coll. [17] and confirmed by us, for both monomolecular (**TEL21/Na^+^**) [18] and tetramolecular **(TG4T)_4_/Na^+^** [19] **G**-quadruplexes deprotonation of radical cations gives rise to (**G**-H2)^•^ radicals because H1 protons participate in Hoogsteen hydrogen bonds (Figure 1b). Moreover, these studies evidenced that deprotonation is much slower compared to other DNA systems, for which it occurs on the ns time-scale [11,13]. Accordingly, the broad absorption band present in the 3 µs spectrum was attributed to a mixture of the **G** radical cation and the (**G**-H2)^•^ radical. The peak at 600 nm is still present at 10 ms (Figure 8b; see also normalized spectra in Figure S2). This contrasts with the behavior of the two previously studied **G**-quadruplexes **TEL21/Na^+^** and **(TG4T)_4_/Na^+^**, for which complete (**G**-H2)^•^ → (**G**-H1)^•^ tautomerisation has already occurred at this time. However, it cannot be ruled out that a small population of (**G**-H1)^•^ radicals is also present. The problem is that the spectrum below 500 nm is dominated by an unknown photoproduct, which does not stem from radicals, as attested by the dependence of the decays on the excitation intensity (Figure S4). Its fingerprint is also present in the steady-state differential absorption spectra recorded before and after irradiation (Figure S3).

For a quantitative description of the radical population, we determined the concentration of hydrated ejected electrons [e_hyd_^−^]_0_ produced by the same excitation intensity as that used for recording the transient spectra in Figure 8 (15.6 × 10^−7^ mol L^−1^). Subsequently, we represented the transient spectrum recorded at 3 µs on ΔA/[e^−^]_0_ scale, (Figure 8a) and reconstructed the broad absorption band in the visible spectral range by linear combinations of the (**G**)^•+^ [10] and (**G**-H2)^•^ [16] spectra, reported for monomeric guanosines. The best agreement in the 450–700 nm area is obtained for combinations 45 (±2)% of (**G**)^•+^ with 55 (±2)% for (**G**-H2)^•^. The lower intensity found for the **G**-quadruplex spectrum around 400 nm is explained by the fact that the radical cation in **G**-quadruplexes absorbs less than the mono-nucleotide dGMP, while at 500 nm the molar absorption coefficient is practically the same [18]. Moreover, the differential absorbance of **TEL25/Na^+^** of the UV band is lower because its ground state absorption is stronger than that of dGMP, as shown in the inset of Figure 8 (see also Figure S5). The radical cation population surviving at 3 µs (45%) is quite close to what was found for **TEL21/Na^+^**(50%) [18] but significantly higher compared to **(TG4T)_4_/Na^+^** (25%) [19].

**Figure 9:**
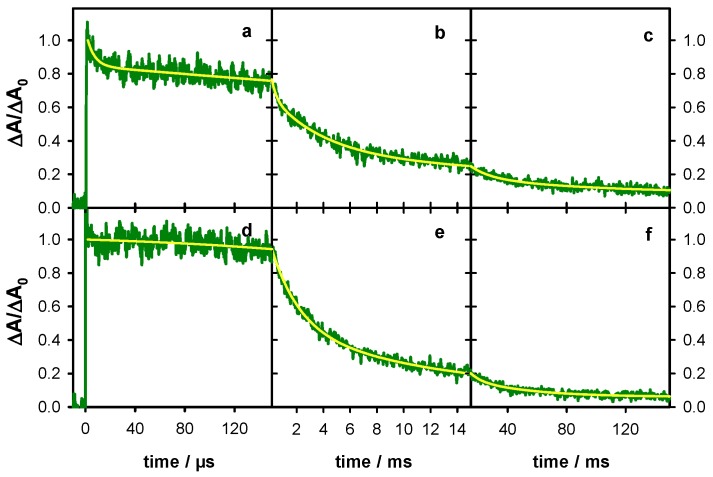
The transient absorption traces recorded for **TEL25/Na^+^** (green) at 500 nm (**a**,**b**,**c**) and 605 nm (**d**,**e**,**f**). The yellow lines correspond to fits with the bi-exponential or tri-exponential functions. For all signals, the absorbance at 2 µs (ΔA_0_) was set equal to 1.

Based on the spectrum at 0.5 ms (Figure 8b) and using a molar absorption coefficient of 2100 mol^−1^ L cm^−1^ at 600 nm, determined for monomeric (**G**-H2)^•^ radicals [10,16], we found that their concentration corresponds to 60 ± 2% of the initial radical concentration. This means that ~40% of the radical cations reacted between 0.5 µs and 0.5 ms, through a process other than deprotonation to (**G**-H2)^•^. This is also reflected in the transient absorption signals at 500 nm, dominated by the radical cation and 605 nm dominated by the (**G**-H2)^•^ radical (Figure 9). The former shows a sharp decrease described by a time-constant of 6 µs (Figure 9a), while a concomitant rise cannot be distinguished on the latter (Figure 9d). As expected, the decays on the ms time-scale are wavelength dependent, t_1/2_ being 2.4 and 3.1 ms, at 500 and 605 nm, respectively.

The spectral evolution in Figure 8 and the associated dynamics in Figure 9 greatly differ from those reported previously for **TEL21/Na^+^** [18]. For the G-quadruplex structure formed by the shorter sequence, 50% of the radical cation population deprotonates with a time constant of 1.2 ms instead of 6 µs for the longer one. Moreover, the disappearance of the (**G**-H2)^•^ radical in **TEL21/Na^+^** is concomitant with that of the radical cation, giving rise to (**G**-H1)^•^ radical whose population at 5 ms amounts to 50% of the initial radical population.

## 3. Reaction Schemes of Nucleic Acids

In general, reactions involving radicals of nucleobases are likely to be bi-molecular. For example, the formation of 8-oxodGuo involves a hydration step requiring addition of a water molecule to **G**^+^**^•^**, while that of guanine-thymine adducts requires the attack of thymine to the (**G**)**^•+^** [8,9]. The probability that the reactants come close to each other is not homogeneous over the three-dimensional space but it is determined by the conformation of the nucleic acid, which, in turn, structures the local environment, including the water network [63]. Thus, conformational motions, occurring on the same timescale as the reaction, may have two effects—on the one hand, it may differentiate the behavior of various reaction sites and, on the other, it may modify the behavior of a given site in the course of the observation. As a result, important deviations appear from the classical models widely used to describe kinetics of chemical reactions in homogenous solutions. The underlying assumption in such models is that of a well-stirred chemical reactor, which means that at the time scale of the observation, there is an internal averaging of all the reaction sites, randomly distributed in three dimensions. The lack of these conditions leads to multiscale decay patterns and renders the notion of rate constant inappropriate, with the reaction rate being time dependent. A good illustration of such multiscale dynamics in DNA is provided by the relaxation of the electronic excited states in helical structures, involving interactions among nucleobases, which spans over, at least, five decades of time [64,65,66]. In general, the description of inhomogeneous dynamical processes necessitates specific theoretical treatments and/or simulations, developed in various fields such as photocatalysis, charge and energy transport in restricted geometries, polymerization reactions, reactions in biological cells, etc. (see for example references [67,68,69,70,71]).

Given the above considerations, the fits of the transient absorption signals with multi-exponential decays presented in Figure 6 and Figure 9 are, in principle, devoid of physical meaning. We simply used the fitted functions for a quantitative description of the decays, allowing easier comparison among the dynamics of the various systems (Figure 7, Table 1). However, in the case of **TEL25/Na^+^**, we refer to a time constant of 6 µs (Figure 9a). Although the exponential nature of this decay is certainly an approximation, its association with changes observed in the time-resolved spectra (Figure 8) and the quantification of the radical population allowed the assigning of this characteristic time to the reaction of approximately 45% of the population of initially created radical cations, while the other 55% reacted much faster.

Coming to the comparison of our results with those reported for the same systems using oxidation by sulfate radical ions, there is one common point. Our transient spectra recorded for **D** at 5 µs are in agreement with those reported in reference [28], as attested by a few comparative points also shown in Figure 4. However, the spectra of **TEL25/Na^+^** obtained by the two methods are in stark contrast—we detected a spectral evolution which is correlated in a quantitative way to (**G**)**^•+^** and (**G**-H2)^•^ radicals. The study performed via the indirect approach did not reveal any time-dependence of the spectra, which were attributed to (**G**-H1)^•^ radicals [29]. Most dramatic divergences appear in the dynamics. In Table 1, the half times determined for the studied systems by the two approaches are shown. In all cases, the t_1/2_ values found from direct photoinonization are significantly shorter. The largest difference is encountered for the single strands for which the t_1/2_ values reported in indirect oxidation studies are more than one order of magnitude larger than those found by photoionization.

The discrepancies between the results obtained by the two methods could be explained by the non-classical reaction schemes discussed above, involved in radical generation.

In direct photoionization, radicals are formed in zero time in respect to our time resolution. At the earliest time that the spectra of radicals can be recorded (2–3 µs), we found that their concentration equals that of the observed ejected electrons. Thus, we were able to follow the fate of the entire radical population, even if part of the deprotonation process was missed.

In the indirect approach, the laser induced reaction occurring at zero time is the production of sulfate radicals (Na_2_S_2_O_8_ → SO_4_^•−^), while the charge transfer reaction with DNA is a diffusion controlled bi-molecular process [72]. As the nucleobase undergoing the oxidation is not necessarily a guanine [72], there are potentially 30 electron donating sites per single strand, 60 per duplex and 25 per **G**-quadruplex. The occurrence of many spatially correlated electron donors, renders the reaction scheme highly inhomogeneous. In addition, nucleic acids are negatively charged electrolytes making the approach of a negatively charged donor particularly selective. Thus, it would not be surprising that the formation of radicals is not limited in a few µs, on which the corresponding transient absorption exhibits a clear rise (Figure 3 in reference [29]). This fast rise, which has been correlated to a reaction rate, may simply concern only part of the sulfate radicals located in positions favoring the reaction, while other sulfate radicals react on longer times. A simple estimation of the sulfate radical concentration produced under the described experimental conditions (13 × 10^−6^ mol L^−1^) shows that it is indeed twice as high as **G** radicals (6.7 × 10^−6^ mol L^−1^). Details are given in the SI. However, it is also possible that non-homogenous diffusion controlled reactions is not the only reason for the longer transient absorption decays found via indirect oxidation. As a matter of fact, the 1 s spectrum reported in Figure 2 in reference [28] clearly differs from those at 5 µs and 10 ms corresponding to **G** radicals. It could be due, for example, to species resulting from reactions of **G** radicals with impurities present in Na_2_S_2_O_8_. Considering that impurities in analytical grade chemicals may reach 1–2%, their concentration in the studied solutions could be two orders of magnitude higher than that of **G** radicals (see SI).

In photoionization experiments, the DNA solutions contain no additives which may react with radicals and shorten their lifetimes. In order to check if the phosphate buffer, which scavenges the produced hydrated electrons, gave rise to secondary reactions, we: (i) diluted it by a factor of 10; and (ii) replaced it by a NaCl solution with the same ionic strength, but none of these modifications altered the dynamics. Along the same line, it was found that, within the precision of our measurements, neither the population nor the decays of deprotonated radicals are affected by oxygen, air equilibrated and argon saturated solutions giving the same signals. These observations suggest that the formation of the final reaction products stemming from (**G**-H1)^•^ and (**G**-H2)^•^ radicals involve just water molecules and/or parts of the nucleic acid itself (other nucleobases, 2-deoxyribose moieties).

## 4. Materials and Methods

### 4.1. Spectroscopic Measurements

Steady-state absorption spectra were recorded using a Lambda 850 (Perkin-Elmer) spectrophotometer. The transient absorption setup used as an excitation source for the fourth harmonic of a Nd:YAG laser (Spectra-Physics, Quanta Ray). The excited area at the surface of the sample was 0.6 × 1.0 cm^2^. The analyzing beam, orthogonal to the exciting beam, was provided by a 150 W Xe-arc lamp (Applied Photophysics, OSRAM XBO). Its optical path length through the sample was 1 cm while its thickness was limited to 0.1 cm in order to use the most homogeneous part of the light. It was dispersed in a Jobin-Yvon SPEX 270M monochromator, detected by a Hamamatsu R928 photomultiplier and recorded by a Lecroy Waverunner oscilloscope (6084). For measurements on the sub µs-scale, the Xe-arc lamp was intensified via an electric discharge. Transient absorption spectra were recorded using a wavelength-by-wavelength approach. Fast shutters were placed in the path of both laser and lamp beams, thus, the excitation rate was decreased from 10 Hz to 0.2 Hz. The incident pulse energy at the surface of the sample was measured using a NIST traceable pyroelectric sensor (OPHIR Nova2/PE25). Potential variations during a measurement were monitored by detecting a fraction of the exciting beam by a photodiode. In addition, the absorbance of the naphthalene triplet state, whose quantum yield in cyclohexane is 0.75 [73], served as actinometer.

### 4.2. Sample Preparation and Handling

Lyophilized oligonucleotides, purified by reversed phase HPLC and tested by MALDI-TOF, were purchased from Eurogentec Europe. They were dissolved in a phosphate buffer (0.15 mol L^−1^ NaH_2_PO_4_, 0.15 mol L^−1^ Na_2_HPO_4_), prepared using ultrapure water delivered by a MILLIPORE (Milli-Q Integral) system. The pH, measured by a HANNA Instr. Apparatus (pH 210), was adjusted to 7 by the addition of a concentrated NaOH solution. A dry bath (Eppendorf-ThermoStatplus) was used for thermal treatment. For the formation of double and four-stranded structures, an appropriate mother solution (2 mL) was heated to 96 °C during 5 min, cooled to the melting point of the corresponding system (cooling time: 1 h), where the temperature was maintained for 10 min. Subsequently, the solution was cooled to 4 °C (cooling time: 2 h), where it was incubated overnight. Representative melting curves are shown in Figure S6. The melting points, found for **D** and **TEL25/Na^+^** are, respectively, 76 °C and 62 °C.

Oligonucleotide solutions were kept at −20 °C. Prior to time-resolved experiments, the sample (2 mL contained in a 1 cm × 1 cm QZ cell) was mildly stirred and its temperature was maintained at 23 ± 0.5 °C. We checked that the stirring did not artificially shorten the decays by cutting it off during each measurement. The absorbance on the excitation side was 0.25 ± 0.02 over 0.1 cm, corresponding to concentrations of approximately 1 × 10^−5^ mol L^−1^, 5 × 10^−6^ mol L^−1^ and 1.2 × 10^−5^ mol L^−1^, respectively for single, double and four-stranded systems. These values are at least one order of magnitude higher than the concentration of ejected hydrated electrons. At each wavelength, a series of three successive signals, resulting from 20–50 laser shots each, were recorded. If judged to be reproducible, they were averaged to reduce the signal-to-noise ratio.

## 5. Conclusions

The present study on **G** radicals, formed by direct photoionization of nucleic acids using low intensity laser pulses, brought new insights and raised new questions regarding radical generation and reactivity in nucleic acids. Below, we focus on a few points which deserve attention in respect to future developments.

In line with previous studies, it was found that **G**-quadruplexes exhibit a larger propensity than duplexes to photoeject an electron upon absorption of low energy photons. One hypothesis suggested previously is that electron ejection occurs after population of excited charge transfer states involving different bases, followed by charge separation [74]. According to such a scenario, the guanine core should behave as a deep trap for the positive charge, the negative charge remaining on an external base (adenine or thymine). This could explain our observation that, going from **TEL21/Na^+^** to **TEL25/Na^+^** by the addition of TA and TT steps at the two ends of the telomeric sequence, the quantum yield of one photon ionization at 266 nm increases from 4.5 × 10 ^−3^ to 5.2 × 10 ^−3^. A systematic study of **G**-quadruplexes with carefully chosen flanking groups and loops could contribute to check the validity of the above mentioned mechanism.

Our methodology, allowing the determination of populations of the various types of radicals in respect to the ejected electrons, showed that the most important part of radical cations undergoes deprotonation. The lifetime of deprotonated radicals is independent of external conditions (phosphate buffer, oxygen, excitation intensity) but it does depend on the base sequence forming a given secondary structure. This behavior suggests that guanine radicals react internally, with other parts of the nucleic acid and/or water molecules participating in the local structure [63,75]. However, we found no indication in the literature about DNA lesions issued from internal reactions of **G** deprotonated radicals or for any reaction involving (**G**-H2)^•^ radicals. Molecular modeling [76] will certainly help understanding such radical reactions.

For all types of radicals, the dynamics deviates from classical reaction kinetics describing monomolecular and bimolecular reactions that take place in homogenous three-dimensional environment. This deviation may also interfere in studies of **G** radicals formed indirectly by the mediation of an oxidant. When the oxidation step involves diffusion of the reactants, the long-time behavior of radicals may be blurred by delayed oxidation due to non-homogeneous, and, therefore, multiscale reactions. However, such indirect studies, if they are limited to early times may bring precious information. This is the case, for example, of the work by Su et al. [17], which managed to grasp important features of radical cations in G-quadruplexes.

## Figures and Tables

**Table 1:** Half-times (in ms) at which the intensity of the transient absorbance signals is decreased by a factor of 2.

Method	S1	S2	D	TEL25/Na^+^
direct photoionization	1.8 ^1^	2.2 ^1^	4 ^1^	2.4 ^1/^3.1 ^2^
indirect oxidation	120 ^3^ [28]	40 ^3^ [28]	7.5 ^3^ [28]	8 ^3^ [29]

^1^ 500 nm; ^2^ 605 nm; ^3^ 510 nm.

## Supplementary Materials

The following are available online, Figure S1: Dependence of the radical decays in **S1** on the excitation intensity, Figure S2: Post-irradiation steady-state differential spectra of **TEL25/Na^+^**, Figure S3: Normalized transient absorption spectra of **TEL25/Na^+^**, Figure S4: Dependence of the radical decays in **TEL25/Na^+^** on the excitation intensity, Figure S5: Steady-state absorption spectra of **D** and **TEL25/Na^+^**, Figure S6: melting curves of **D** and **TEL25/Na^+^**, Estimation of radical concentrations in reference [28].
