# Supplementary material for: Populations and Dynamics of Guanine Radicals in DNA strands—Direct versus Indirect Generation
Source: Molecules. 2019 Jun 26;24(13):2347. doi: 10.3390/molecules24132347 (PMC6651618; doi:10.3390/molecules24132347)
Supplement: Supplementary file 1 [file molecules-24-02347-s001.pdf]

Populations Dynamics of Guanine Radicals in DNA strands - Direct versus Indirect Generation

Evangelos Balanikas [1], Akos Banyasz [1,2], Gérard Baldacchino[1] and Dimitra Markovitsi[1,*]

[1] LIDYL, CEA, CNRS, Université Paris-Saclay, F-91191 Gif-sur-Yvette, France
vangelis.balanikas@cea.fr (E.V.); gerard.baldacchino@cea.fr (G.B.); dimitra.markovitsi@cea.fr (D.M.)
[2] Univ Lyon, ENS de Lyon, CNRS UMR 5182, Université Claude Bernard Lyon 1, Laboratoire de Chimie, F-69342 Lyon, France; akos.banyasz@ens-lyon.fr (A.B.)
* Correspondence: dimitra.markovitsi@cea.fr (D.M.)

Supporting Information

- **Figure S1: Dependence of the radical decays in S1 on the excitation intensity**
- **Figure S2: post-irradiation steady-state differential spectra of TEL25/Na$^+$**
- **Figure S3: normalized transient absorption spectra of TEL25/Na$^+$**
- **Figure S4: Dependence of the radical decays in TEL25/Na$^+$ on the excitation intensity**
- **Figurer S5: steady-state absorption spectra of D and TEL25/Na$^+$**
- **Figure S6: melting curves of D and TEL25/Na$^+$**
- **Estimation of the G radical and $SO_4^{\bullet-}$ concentrations in reference [1].**

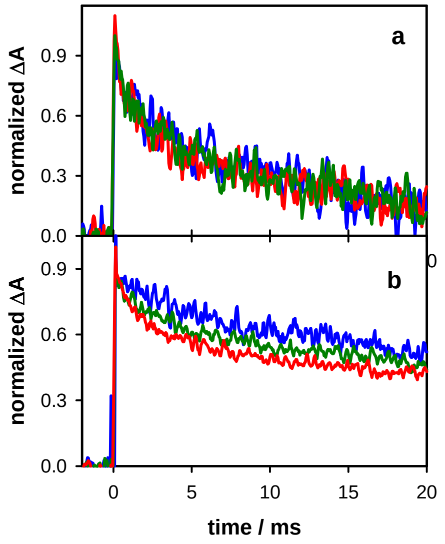

Figure S1. Normalized transient absorption signals recorded for **S1** at 500 nm (**a**) and 305 nm (**b**) for excitation energies of 4 mJ (blue), 6 mJ (green) and 7 mJ (red).

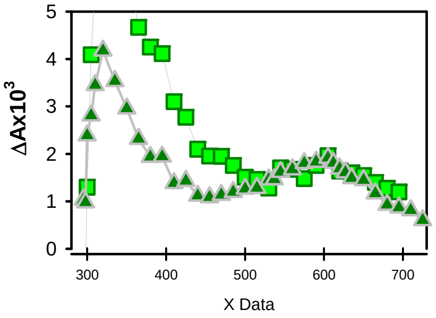

Figure S2. Differential absorption spectra recorded for **TEL25/Na$^+$** at 0.5 ms (triangles) and 10 ms (squares; its intensity was normalized to that of 0.5 μs the spectrum at 600 nm).

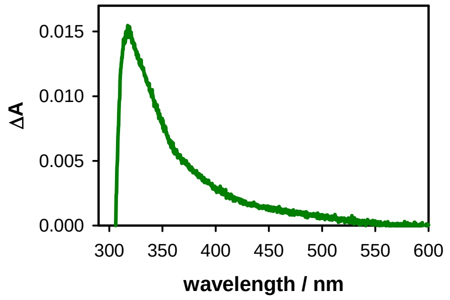

Figure S3. Differential steady-state spectrum corresponding to the absorbance of **TEL25/Na$^+$** before and after irradiation with 400 laser pulses of 6 mJ.

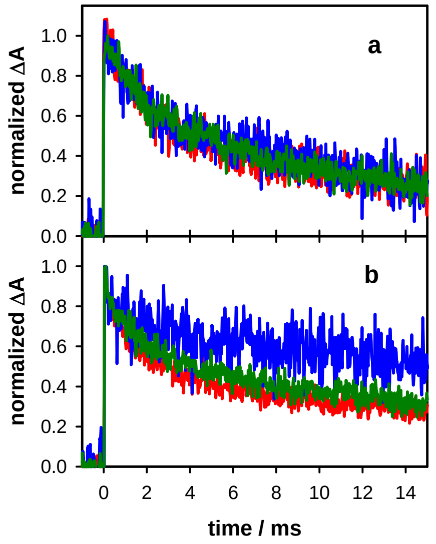

Figure S4. Normalized transient absorption signals recorded for **TEL25/Na⁺** at 605 (**a**) and 305 nm (**b**) for excitation energies of 2 mJ (blue), 4mJ (green) and 6 mJ (red).

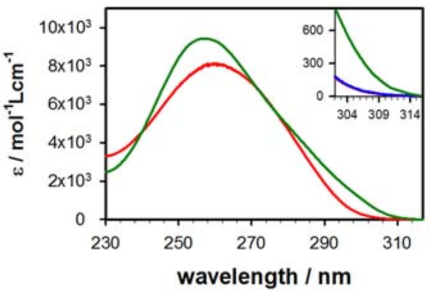

Figure S5. Absorption spectra of **D** (red) and **TEL25/Na⁺** (green). The molar absorption coefficients ε were estimated, starting from the ε_{260nm} values provided by Eurogentec Europe for single strands at room temperature. In the case of **D**, we assumed that ε_{260nm} at 96°C corresponds to that of an equimolar mixture of **S1** and **S2** at the same temperature. In the case of **TEL25/Na⁺**, we simply considered that the ε_{260nm} value at 96°C is that of the single strand. We judge that the errors due these approximations do not exceed 15%.

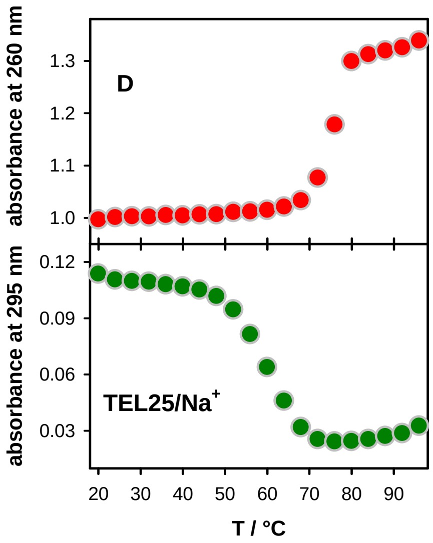

Figure S6. Absorbance variation determined as a function of temperature for the duplex **D** at 260 nm and the **G**-quadruplex **TEL25/Na+** at 295 nm.

Estimation of the G-radical and $SO_4^{\bullet -}$ concentrations reactions in reference [1]:

The radical concentration, evaluated using a molar absorption of 1500 $mol^{-1}cmL^{-1}$ at 500 nm [2] and an absorbance of 0.01 (Figure 3 in reference [1]) is 6.7×10^{-6} $molL^{-1}$. We estimated $[SO4^{\bullet -}]_0$ considering the excitation energy (60 mJ), the excitation path length (0.2 cm), the excited volume (0.06 mL) and the $Na_2S_2O_8$ concentration (0.02 $molL^{-1}$) reported by the authors and taking into account the molar absorption coefficient of $Na_2S_2O_8$ at 308 nm (1 $mol^{-1}Lcm^{-1}$) and the quantum yield for $SO4^{\bullet -}$ formation (0.55) [3]. The resulting concentration, 13×10^{-6} $molL^{-1}$, is twice as high as that of **G** radicals.

($Na_2S_2O_8$): 0.02 $molL^{-1}$
Excitation path length: 0.2 cm
Molar absorption coefficient at 308 nm: 1 $mol^{-1}Lcm^{-1}$
Absorbance at 308 nm: 0.004
Incident excitation energy: 60 mJ
Absorbed excitation energy: $60\times(1-10^{-0.004})=0.55$ mJ
Absorbed photons: 1.4×10^{-9} einstein
Quantum yield of the reaction: 0.55
$SO_4^{\bullet -}$ formed par laser pulse: 7.7×10^{-10} mol
Excited volume: 0.06 mL
$SO_4^{\bullet -}$ concentration: $1.3x10^{-5}$ $molL^{-1}$

References

1. Rokhlenko, Y.; Cadet, J.; Geacintov, N. E.; Shafirovich, V., Mechanistic Aspects of Hydration of Guanine Radical Cations in DNA. *J. Am. Chem. Soc.* **2014,** 136, (16), 5956-5962.

2. Candeias, L. P.; Steenken, S., Stucture and acid-base properties of one-electron-oxidized deoxyguanosine, guanosine, and 1-methylguanosine. *J. Am. Chem. Soc.* **1989,** 111, 1094-1099.

3. Heidt, L. J.; Mann, J. B.; Schneider, H. R., The photolysis of persulfate. 2. The quanrum yield in water and the effect of sodium chloride in dilute alkaline solution. *J. Am. Chem. Soc.* **1948,** 70, (9), 3011-3015.
